# Development of a new guideline to facilitate diagnosis and management of rib stress injuries in rowers

**DOI:** 10.1186/2052-1847-7-S1-O3

**Published:** 2015-08-11

**Authors:** Guy Evans, Ann Redgrave

**Affiliations:** 1 GB Rowing Team, British Rowing, 6 Lower Mall, Hammersmith, London, W6 9DJ

## Background

Rib stress injury is the development of pain due to bone oedema caused by overload along the rib shaft and is commonly seen in rowers [1,2]. At the elite level the injury is often managed by clinicians experienced with the condition. However it has been noted by the authors that there is a lack of awareness and confidence in diagnosing and managing this condition by clinicians who are not regularly exposed to this mainly rowing specific injury. As a result, a guideline has been developed by the authors to aid diagnosis and management of rib stress injury for use by clinicians.

## Materials and methods

A detailed literature search was conducted reviewing the diagnosis and management of rib stress injury. Detailed discussions between the authors and the rest of the Great Britain rowing medical team took place to highlight the key issues in treating rib stress injury. An up-to-date and evidence based approach to managing rib stress injury was created using both expert knowledge and the current literature.

## Results

The outcome from the expert meetings and literature review was the creation of a new guideline for management of rib stress injuries. The guideline was created to be user friendly, informative and logical and to direct the reader through diagnosis, investigations and management of rib stress injury. A separate section is included in the guidance which helps identify both intrinsic and extrinsic factors that may predispose to injury. The guideline has deliberately been kept to two pages long so as not to overwhelm the reader or complicate the guidance.

## Conclusions

A new clinical guideline for management of rib stress injuries has been developed to facilitate clinicians and in identifying rib stress injury and aid accurate diagnosis and management. This guideline is to be disseminated to clinicians, rowing coaches and clubs throughout the UK.
